# Cognitive loopholes of crime: Mapping the Codevelopment of moral disengagement within perceptions of risks and rewards

**DOI:** 10.1111/jora.70056

**Published:** 2025-07-29

**Authors:** Romain Decrop, Bri McManamon, Kaylie Williams, Kerry Houlihan, Haely Crouch Bok, Kaelynn Knestrick, Emma Rodgers, Meagan Docherty, Elizabeth Cauffman

**Affiliations:** ^1^ University of California Irvine California USA; ^2^ Bowling Green State University Bowling Green Ohio USA

**Keywords:** crime, moral disengagement, perception, punishment, reward, risk

## Abstract

Prior research has examined individuals' perceptions of punishments (PP) and rewards (PR) for crime, as well as their use of moral disengagement (MD), to understand why adolescents and young adults commit crimes. However, the joint development of these cognitions as a broader risk‐perception mechanism remains understudied. This paper explores the independent and relational development of these processes in justice‐involved youth. Data from 1,170 male participants (42.1% Black, 34.0% Hispanic, 19.2% White, 4.6% Other) in the Pathways to Desistance study were analyzed using a three‐variable autoregressive latent trajectory model. MD, PP, and PR were measured across 11 waves and 7 years, allowing for the simultaneous examination of individual trajectories and their bidirectional relationships from adolescence to young adulthood. Although PP increased and MD and PR decreased across adolescence, all three exhibited decelerations in their change prior to young adulthood. Moreover, bidirectional relationships between the processes suggest the presence of harmful developmental cycles that may prematurely halt justice‐involved youths' cognitive growth related to risk perception. Findings suggest that distorted risk and reward perceptions of crime, amplified by MD, may create harmful developmental cycles during adolescence that distort risk perception in adulthood. Further, the decelerations from late adolescence to young adulthood (~ages 18–22) point to a salient critical transitional period of development for these processes. These results may help inform developmentally tailored programs for at‐risk youth. By targeting PP, PR, and MD as intertwined processes, interventions may recalibrate maladaptive perceptions, disrupt risky decision‐making cycles, and reduce long‐term offending.

## INTRODUCTION

The societal cost of crime has engendered over a century of research aimed at identifying psychosocial factors that predict harmful behaviors (Baskin‐Sommers et al., 2015; Healy & Bronner, 1916). These efforts have identified middle adolescence to young adulthood as the period of greatest risk for engaging in crime despite risk awareness (i.e., age–crime curve; Stander et al., 2023). This is in part due to underdeveloped self‐regulatory systems and morals, as well as a heightened drive for reward stemming from peaks in reward sensitivity (Bandura et al., 1996; Shulman & Cauffman, 2013). Thus, to better understand risk perception in adolescents and young adults, research has focused on cognitive factors that impact risky decision‐making mechanisms (perceptions of punishments (PP) and rewards (PR); moral disengagement (MD)) to identify and treat individuals at risk of engaging in crime. However, prior work has generally considered these factors individually, overlooking how they may be intertwined and working together as part of a broader mechanism across development. This important nuance can help shed light on changes in overarching thought processes that are involved in the decision to commit a crime.

### Theoretical background

The rational theory of crime suggests that individuals weigh the immediate rewards of misconduct against the potential for punishment when deciding whether to offend (Beaudry‐Cyr, 2015). In other words, the decision to engage in crime is informed by the cooccurring cognitive processes of punishment and reward perception. PP are an individual's beliefs of personal, social, and legal punishments that can result from engaging in crime (Apel, 2013), while PR describe one's expectations of social and personal rewards for committing crime (Reyna & Farley, 2006). Indeed, empirical work has shown that adolescents and young adults with higher reward and/or lower punishment sensitivity are more likely to offend (e.g., Morgan et al., 2014; Shulman et al., 2017).

Simultaneously, moral theories posit that individuals avoid behaviors that may result in negative self‐evaluation, since recognition that one's behavior is morally transgressive can lead to unpleasant internal feelings (e.g., guilt; Festinger, 1957). Thus, to maintain a positive self‐image, some individuals use cognitive strategies to mitigate the negative affect associated with immoral behaviors. Specifically, MD targets the decision‐making process by justifying or rationalizing behaviors that violate moral standards (Bandura, 2016). Bandura (2011, 2016) argued that individuals first diminish the severity of their detrimental practice (e.g., stealing is wrong) by employing *moral justifications* (e.g., “Stealing fights corporate greed”), *euphemistic language* (e.g., “I'm just borrowing it”), and/or *advantageous comparisons* (e.g., “At least it's not murder”). Then, they may mitigate the injurious effects of the act by *distorting the consequences* (e.g., “It wasn't that bad”) and *displacing or diffusing their responsibility* to minimize their personal accountability (e.g., “I was following orders”, “Everyone was doing it”). Finally, if confronted with the transgression, some may rely on *attributions of blame* (e.g., “They brought it on themselves”) or *dehumanization* (e.g., viewing others as less than human) to reduce their victim related guilt. Unsurprisingly, the use of MD has also been linked to offending in both adolescent and young adult samples (Cardwell et al., 2015; Shulman et al., 2011).

Beyond their independent functions, moral justifications and evaluations of PP and PR converge, as moral factors shape risk and reward assessments, sometimes overriding rational decision‐making (Pogarsky, 2002; Wikström et al., 2017). Broadly, the tripartite theory suggests that individuals consider the expected utility (i.e., obtained outcome; PR) along with both the external (i.e., consequences, risk; PP) and internal disutility (e.g., guilt, remorse; morality processes) of their transgression (Hart & Curtis, 2023). Notably, while younger children initially prioritize expected rewards, brain maturation through adolescence and young adulthood fosters a deeper consideration of the consequences and guilt associated with their actions (Decrop & Docherty, 2024). These findings and developmental shifts align with Neo‐Kohlbergian theories, where an early reliance on external PP and PR processes transitions toward internalized social norms and moral principles (Rest et al., 1999), and underscore how moral maturation can reshape youths' evaluations of punishments, rewards, and justifications. However, despite evidence that the PP, PR, and MD processes individually influence criminal decision‐making during adolescence and young adulthood (Moore, 2015; Shulman et al., 2011; Walters, 2018), little empirical work has examined how these three processes codevelop as a broader crime decision‐making mechanism.

This study explores the three‐way bidirectional development of PP, PR, and MD in a justice‐involved sample of youth to illustrate why some individuals may choose to offend across the age‐crime curve. This population is particularly important to study given its heightened risk for long‐term patterns of harmful behaviors (e.g., offending, substance use) and resulting need for intervention. Our results inform theory across disciplines (e.g., cognition, criminology, development) and will help guide the creation of developmentally appropriate programs that target PP, PR, and MD to reduce long‐term risk taking, antisocial behavior, and crime.

### Punishment, reward, and moral disengagement: Individual growth

The dual systems model proposes that adolescents take risks due to asynchronous brain development, in which limbic and reward‐processing regions (e.g., nucleus accumbens) mature early in adolescence, while the prefrontal cortex, which is responsible for executive functioning skills such as long‐term planning and impulse control, does so through young adulthood (Galvan et al., 2006, 2007; Qu et al., 2015). As a result, younger teens' heightened reward sensitivity makes them more likely to be impulsively reward‐driven and to underestimate the potential consequences of their actions. This effect can be amplified by peaks in peer orientation, when peer approval is highly valued, making both social and personal rewards (e.g., thrill) especially salient to consider across adolescence (Farrington, 2010; Steinberg, 2008). Over time, as neural connections become more ingrained, psychosocial maturity and cognitive abilities improve, and real‐world experiences shape youths' understanding of punishment, PP tend to increase and PR decrease, with stabilizations in early adulthood as punishments are viewed to be more certain and impactful to adult responsibilities (e.g., work, family; Blakemore & Robbins, 2012; Cauffman & Steinberg, 2000; Piquero et al., 2003). Importantly, among justice‐involved youth, PP increases and PR decreases have been linked to reductions in criminal behavior (Loughran et al., 2012).

Similarly, MD declines across adolescence and young adulthood, and these reductions correspond with less offending in justice‐involved youth (Caprara et al., 2014; Shulman et al., 2011). Within Kohlberg's Theory of Moral Development, MD's peaks in middle adolescence align with conventional stages when there is strong social motivation to make cognitive justifications for harmful behavior (i.e., youth's morality operates on social favorability, making justification of behaviors not aligned with moral values important; Kohlberg, 1981; Paciello et al., 2008). On a cognitive level, this peak likely reflects ongoing growth in prefrontal regions tied to cognitive control, perspective‐taking, and moral reasoning (e.g., dorsolateral & orbitofrontal cortex), as well as weak connectivity between these regions and limbic structures governing emotional processing and empathy (e.g., amygdala, anterior cingulate cortex; Decety & Cowell, 2014; Galvan et al., 2007). As these networks mature through early adulthood (ages 18–25), cognitive and moral judgments improve, and reliance on MD declines (Hyde et al., 2010; Moore et al., 2012). Theoretically, moral justifications may wane with deeper ethical reasoning skills, euphemistic language may diminish with advancements in vocabulary understanding, displacement and diffusion of responsibilities may subdue with greater accountability, and advantageous comparisons, distortion of consequences, attribution of blame, and dehumanization may decline with gains in social awareness, empathy, and cognitive control (Moore, 2015).

Collectively, the development of PP, PR, and MD is shaped by neurocognitive growth and psychosocial experiences. Adolescents are more likely to disengage morally, downplay risks, and prioritize short‐term rewards, while young adults adopt more stable moral frameworks, heightened risk sensitivity, and reduced reward focus. However, no work has simultaneously accounted for within‐ and between‐subject differences to distinguish stable individual differences from time‐varying changes in these mechanisms. Specifically, no research has parsed how people consistently differ from each other (e.g., some always perceive more reward) from the changes that happen within a person over time (e.g., some perceive fewer rewards as they age). This could clarify how these relationships evolve during the normative desistance period of offending and may help create targeted intervention strategies to support at‐risk youth at specific points of their development.

### Punishment, reward, and moral disengagement: Joint growth

Beyond understanding how a given process may change over time on its own, it is of equal importance to understand how the trajectory of one variable may impact the trajectory of another and how the change in one variable predicts the change of another. By examining the joint growth of PP, PR, and MD, we can uncover how these processes reinforce or counteract one another, providing a more integrated and real‐world understanding of the multiple cognitive processes underlying risk perception and criminal behavior. Clarifying these connections across development can help disentangle the complex, time‐dependent relationships among PP, PR, and MD, and can provide insights for designing targeted interventions that disrupt harmful cycles and promote healthy growth. This is especially important because these variables operate similarly within the tripartite theory and may work in tandem to predict salient outcomes. However, little is known about how these factors impact each other across adolescence and young adulthood.

Overall, youth who expect harsher punishments for crimes also tend to view them as less rewarding (Altikriti & Nedelec, 2020). Nevertheless, the bidirectional growth of these factors (i.e., how PP values at one timepoint predict PR values at a subsequent point, and vice‐versa), and whether the associations change as processes mature, remain unexplored. For example, due to deficits in self‐regulation, working memory, and cognitive processing, youth may not take the time, or have the ability, to consider everything associated with the utility and disutility of crime (i.e., cognitive load is too big). Thus, if PP decreases and PR increases after a youth gets away with a crime, the extra focus on the decreased punishment and increased rewards may remove more objective perceptions from consideration. In other words, as risky thoughts about engaging in a crime enter consciousness, less room is allotted for protective thoughts against it. Over time, this may create a cyclical problem, where those with lower PP spend more time thinking about the potential rewards of crime (i.e., increases PR), which then takes focus away from potential consequences (i.e., lowers PP), and ultimately molds distorted PP and PR processes.

Moreover, few studies have developmentally considered how MD is intertwined within this relationship. Notably, Altikriti (2020) found that improvements in PP and PR only reduced offending when accompanied by lower levels of MD. Hypothetically, not only would MD nullify or mitigate the internal disutility of a crime (i.e., reduce guilt), the strategies may also decrease individuals' PP and increase their PR. For example, by downplaying the severity of an act (e.g., “This store makes millions, I'm not hurting anyone.”), MD justifications could reduce fears of punishment by making crime seem less morally wrong and thus deserving of consequences. Simultaneously, by reducing the guilt associated with a crime, individuals may perceive the benefits as more justified or rewarding. Worrisomely, these same individuals who anticipate less punishment and/or expect more rewards may in turn use MD to rationalize their crimes given that they need fewer reductions in guilt. By needing less MD to effectively disengage, the strategies may become engrained as maladaptive coping and/or problem‐solving habits.

Over time, these patterns may negatively impact the development of neural systems and lead to distorted risk perceptions. As highlighted, these processes share limbic and prefrontal foundations (Decety & Cowell, 2014; Galvan et al., 2007; Qu et al., 2015). In early adolescence, heightened activity in reward‐sensitive limbic areas (nucleus accumbens, amygdala) tends to lower PP and increase PR and MD, while later maturations in prefrontal regions (prefrontal, dorsolateral prefrontal, & orbitofrontal cortex) gradually offset these tendencies with improved cognitive control and reasoning. Critically, reductions not only rely on the development of these individual regions, but also on the stronger connectivity between them. Although our study does not directly assess brain function, recognizing this shared neural architecture helps put into context the intertwined nature of our three processes. Then, by exploring their developmental interconnections, we can offer deeper insight into the mechanisms and reinforcing cycles that exacerbate or mitigate cognitive patterns that sustain, normalize, and/or deter criminal behavior.

### Current study

While PP, PR, and MD have independently been linked to offending, their joint growth as a broader, risky decision‐making mechanism remains understudied. Using an autoregressive latent trajectory (ALT) model, we tracked how changes in one factor predict shifts in the others over time, providing nuanced insights into the dynamic growth of these processes in an at‐risk sample who may not follow typical developmental trends. Indeed, we would normatively expect MD and PR to decline and PP to increase into adulthood, with decelerations that plateau through young adulthood as growth stabilizes. However, we hypothesize that justice‐involved youth may experience developmental delays (i.e., changes occur late) or premature terminations in growth (i.e., plateaus occur early) in part due to maladaptive cycles where higher MD is associated with lower PP and higher PR, which are themselves inversely related. Examining these relationships across peaks and desistance periods of the age‐crime curve can help identify when and how risk perception development goes awry and guide developmentally tailored interventions aimed at reducing long‐term offending and promoting healthy cognitive growth.

## MATERIALS AND METHODS

### Participants

The Pathways to Desistance study is a longitudinal investigation of U.S. justice‐involved youth from Philadelphia, PA (*n =* 605 males) or Phoenix, AZ (*n =* 565 males; Schubert et al., 2004). Participants were 14–18 years old at their baseline interview (*M*_age_ = 16.05, SD = 1.16) and had been charged with a felony or serious nonfelony offense. Consistent with prior trajectory studies (Baskin‐Sommers et al., 2015), only male participants (*n =* 1170) are analyzed given the insufficient female sample size (*n =* 184) for trajectory stability (Nagin, 2005). Our sample is 41.1% Black, 34.0% Hispanic, 19.2% White, and 4.6% other (biracial, Native American).

### Procedures

Study investigators recruited 2,008 individuals, and 67% agreed to participate. Trained researchers led participants through two 2‐hour interviews at baseline, with subsequent follow‐up interviews every 6 months for a 3‐year period and then annually for 4 years. Retention rates across the 11 waves were high (range = 84–94%, *M* = 90%), allowing for analyses that leverage the cohort‐sequential design of the study and span across 13 years from adolescence to young adulthood (ages 14 to 26; for full procedures, see Schubert et al., 2004). The original study was approved by the Institutional Review Boards (IRB) at the universities of principal investigators, while this secondary data analysis was approved by the University of California, Irvine's IRB (#20141706).

### Measures

PP, PR, and MD were measured at each wave. Descriptive information, Cronbach's alphas, and rank‐order stability (i.e., bivariate correlation of consecutive waves) are depicted in Table 1. Additionally, the Pearson correlation coefficients of the three variables at and across waves are available in Table S1. More details on the study, including its methods and measures, can be found at www.pathwaysstudy.pitt.edu.

**TABLE 1:** Descriptive and reliability information across waves.

**Months from baseline**	0	6	12	18	24	30	36	48	60	72	84
Wave	1	2	3	4	5	6	7	8	9	10	11
Mean age	16.05	16.56	17.06	17.53	18.03	18.50	19.02	20.04	21.03	22.04	23.04
**Perception of punishments for crime (range = 0–10)**											
*N*	1169	1090	1086	1057	1059	1060	1055	1040	1029	1004	960
Mean	4.95	5.06	5.15	5.25	5.28	5.34	5.30	5.46	5.57	5.68	5.65
SD	1.82	1.88	1.88	1.91	1.92	1.96	1.91	1.94	1.93	1.89	1.86
*α* of three scales	0.60	0.61	0.63	0.65	0.66	0.64	0.64	0.63	0.61	0.59	0.61
CWC	–	0.50	0.54	0.58	0.53	0.56	0.55	0.57	0.54	0.56	0.56
*α* of 7 certainty (you) items	0.89	0.90	0.90	0.92	0.92	0.93	0.92	0.93	0.93	0.93	0.93
*α* of 7 certainty (other) items	0.82	0.84	0.86	0.87	0.89	0.89	0.89	0.91	0.90	0.92	0.91
*α* of 6 social cost items	0.66	0.74	0.76	0.78	0.78	0.78	0.80	0.80	0.82	0.84	0.85
**Perception of rewards for crime (range = 0–10)**											
*N*	1169	1090	1086	1057	1059	1060	1056	1041	1029	1004	960
Mean	3.17	3.03	2.88	2.82	2.79	2.68	2.69	2.60	2.69	2.49	2.70
SD	1.42	1.50	1.56	1.58	1.60	1.62	1.60	1.58	1.55	1.58	1.46
*α* of four scales	0.74	0.74	0.78	0.80	0.80	0.81	0.81	0.79	0.78	0.80	0.77
CWC	–	0.56	0.56	0.59	0.61	0.62	0.60	0.54	0.55	0.55	0.52
*α* of 5 stealing items	0.74	0.79	0.84	0.85	0.86	0.88	0.88	0.89	0.89	0.90	0.90
*α* of 5 robbery items	0.82	0.86	0.87	0.89	0.89	0.91	0.91	0.91	0.92	0.92	0.93
*α* of 5 fighting items	0.73	0.82	0.85	0.87	0.88	0.89	0.90	0.90	0.89	0.90	0.90
*α* of 7 personal items	0.87	0.90	0.89	0.90	0.90	0.91	0.91	0.91	0.93	0.91	0.91
**Moral disengagement (range = 1–3)**											
*N*	1167	1090	1086	1057	1058	1058	1056	1041	1028	1002	959
Mean	1.63	1.59	1.55	1.52	1.51	1.49	1.48	1.45	1.43	1.40	1.39
SD	0.35	0.37	0.36	0.38	0.37	0.36	0.36	0.35	0.34	0.34	0.35
*α* of 32 items	0.88	0.90	0.90	0.92	0.92	0.92	0.92	0.92	0.92	0.92	0.93
CWC	–	0.59	0.61	0.63	0.63	0.67	0.65	0.59	0.57	0.56	0.59

Abbreviations: *α*, Cronbach's alpha; CWC, consecutive wave correlation coefficient; SD, standard deviation.

#### Certainty of punishment and social costs of crime

We created a PP score by averaging three scales from the Social and Personal Costs and Rewards of Crime indices (Nagin & Paternoster, 1994). Specifically, we included the Certainty of Punishment to You and Others scales, each with seven 11‐point Likert scale items, scored 0 (“*No chance*”) to 10 (“*Absolutely certain to be caught*”), that assess the perceived likelihood of the participant or others being caught and arrested for various crimes (e.g., fighting, robbery). We also included the six 5‐point Likert scale items of the Social Costs of Punishment scale, ranging from “(1) *Not likely*” to “(5) *Very likely*” (e.g., “If the police catch me breaking the law, how likely is it that I would be suspended from school?”). To compute a total score at each wave, we averaged each individual scale, rescaled the Social Costs scale to match the others' 0 to 10 range (i.e., first transforming it to 0–1 by subtracting one and dividing by four, then multiplying by 10), and then took the mean of the three. Although this approach preserves meaningful scale anchors and the relative spacing and distribution of responses to aid interpretability, it does not equate scale variances. Nevertheless, combining perceptions of both the probability and social costs of punishment aims to more ecologically mirror real‐life decision making, with higher scores indicating a greater perceived likelihood of getting caught and receiving harsher punishment.

#### Social and personal rewards of crime

Likewise, we computed a PR score from four scales of the Social and Personal Costs and Rewards for Crime indices (Nagin and Paternoster, 1994). Three Social Rewards of Crime scales (stealing, fighting, robbery) take the mean of five 4‐point Likert scale items ranging from *“(1) Strongly Disagree*” to “(4) *Strongly Agree*” (e.g., “If I take things, other people my age will respect me more”), while the Personal Rewards of Crime scale averages seven 11‐point Likert scale items ranging from “(0) *No fun or kick at all*” to “(10) *A great deal of fun or kick*” (e.g., “How much thrill/rush is it to break into a store or home?”). To calculate a total score at each wave, we similarly rescaled the Social Rewards scale to range from 0 to 10 and averaged the four scales' means. Higher scores indicate more anticipated peer approval and personal thrill for committing crimes.

#### Moral disengagement

MD was assessed with the Mechanisms of Moral Disengagement scale (Bandura et al., 1996), which consists of 32 3‐point Likert scale items (1 “*Disagree*” to 3 “*Agree*”) representing the eight previously discussed MD strategies (e.g., dehumanization: “Some people deserve to be treated like animals”). Nevertheless, psychometric investigations generally support a one‐factor structure (Moore et al., 2012), and prior work with this sample has used a total mean score (e.g., Shulman et al., 2011). Thus, we also used total mean scores at each wave, with higher values indicative of generally more permissive attitudes surrounding morally transgressive actions (i.e., greater MD).

### Analytic plan

Data are publicly available (icpsr.umich.edu/web/NAHDAP/studies/29961) and analyses were run in Mplus (Version 8.8; Muthén & Muthén, 1998–2011). To identify the best‐fitting PP, PR, and MD unconditional latent trajectories across the 11 waves, we compared univariate latent growth curve models with varying shapes of growth (i.e., fixed and random effects that specify an intercept only or no growth, linear change, and/or quadratic change that accelerates or decelerates) using a maximum likelihood robust estimator (MLR; Curran & Hussong, 2003). These analyses assume that repeated observable measures reflect underlying growth trajectories, with intercept, slope, and quadratic factors varying across individuals to account for their differences in initial levels and rates of change. Factor loadings were fixed at 1 for the intercepts, spaced 0 to 1.4 for linear growth, and squared (0 to 1.96) for quadratic change. Of note, despite our best efforts to run the ALT model with data aligned by age, that model did not converge, and thus we elected to run all analyses with data aligned by wave.

**FIGURE 1:**
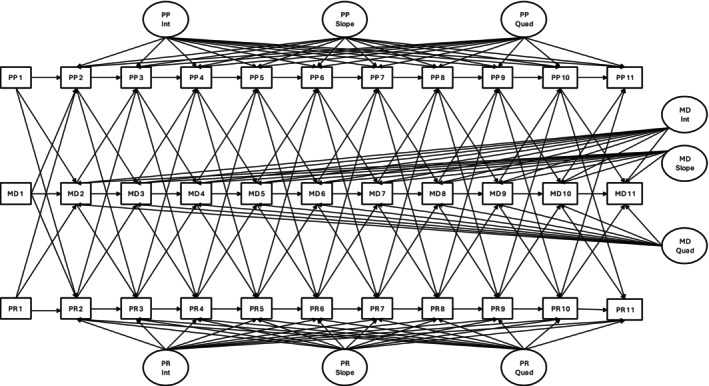
Autoregressive latent trajectory model. Int, intercept growth factor; MD, moral disengagement; pp, perception of punishment for crime; PR, perception of rewards for crime; Quad, quadratic growth factor; Slope, linear growth factor; only regressions shown; correlations omitted for clarity.

The best‐fit models for the three variables were chosen based on lower relative AIC, BIC, SABIC, RMSEA, and SRMR indices, higher relative CFI and TLI indices, and significant chi‐square difference tests of nested models. These growth curves were then integrated into an ALT model to assess their developmental associations (Bollen & Zimmer, 2010). The ALT model leverages the strengths of latent growth curve and autoregressive (cross‐lagged panel) models by estimating both person‐level trajectories and time‐specific, within‐person deviations from those. Thus, the ALT model depicts how long‐term change and short‐term fluctuations in each variable are related, as well as how changes in one may predict changes in the others. To our knowledge, no prior work has published a three‐variable ALT model, making this a novel application of the approach that can be adapted for future longitudinal research (see Figure 1).

## RESULTS

### Punishment, reward, and moral disengagement: Individual growth

#### Latent growth trajectories

**FIGURE 2:**
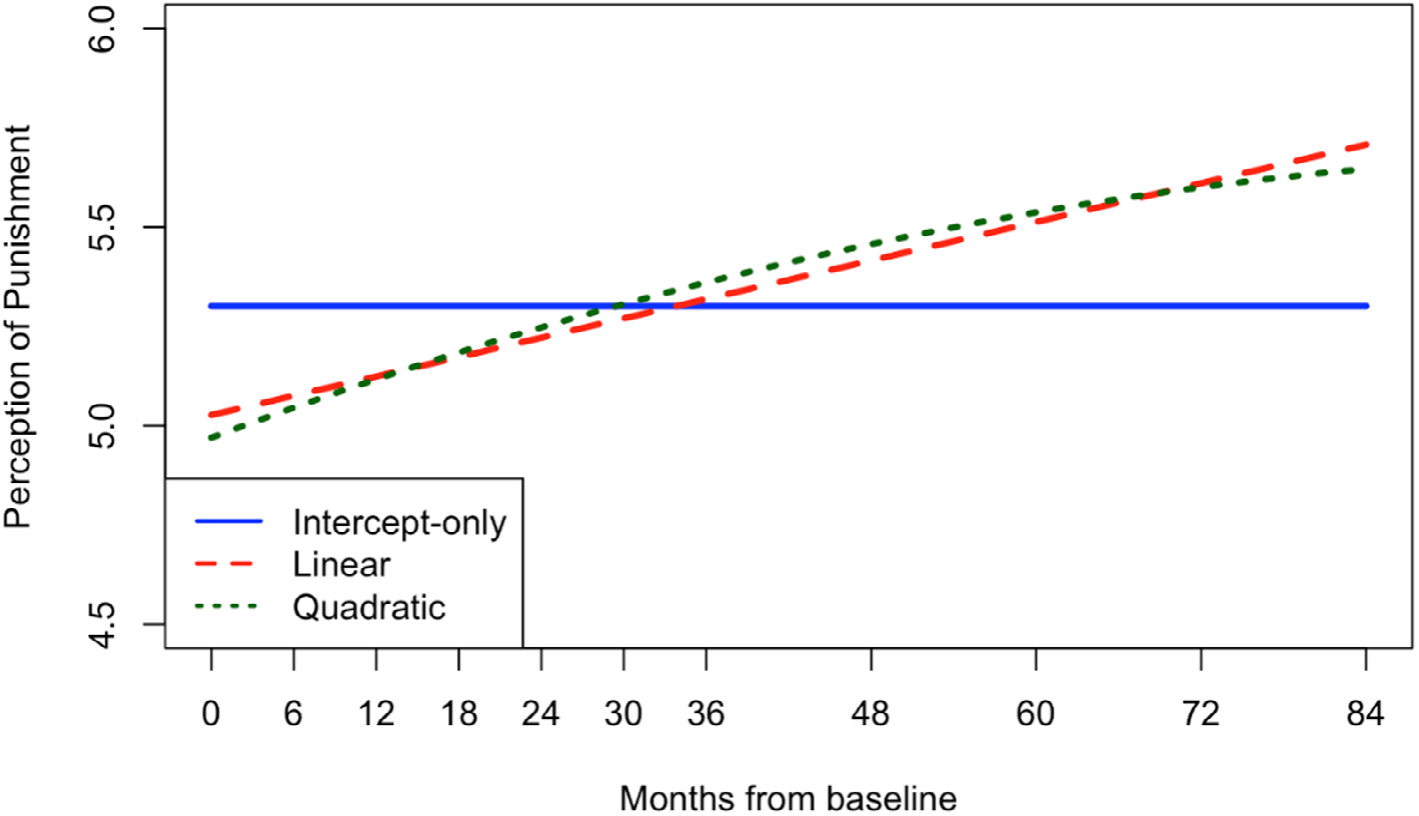
Unconditional latent growth curves of perceptions of punishments for crime.

**FIGURE 3:**
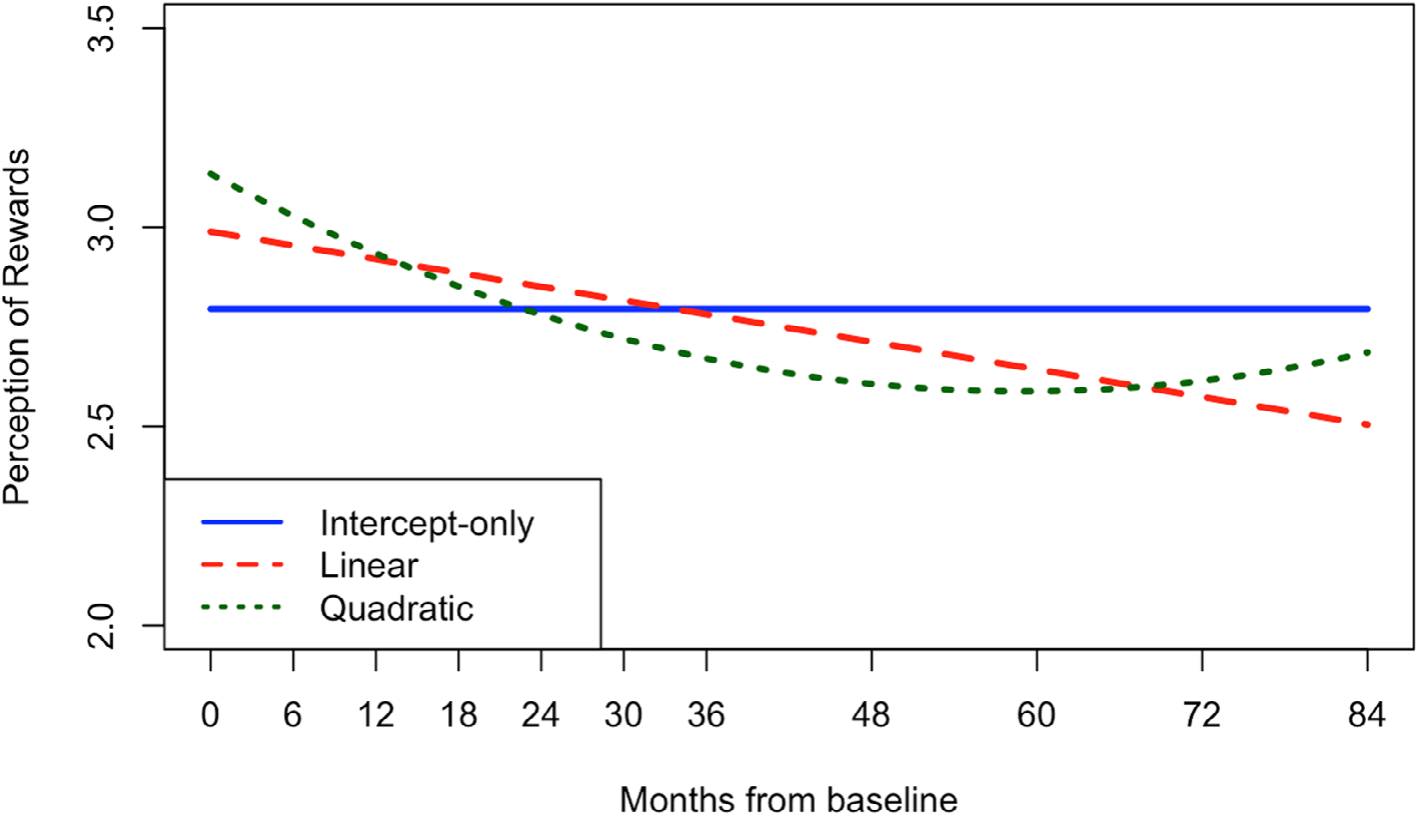
Unconditional latent growth curves of perceptions of rewards for crime.

**FIGURE 4:**
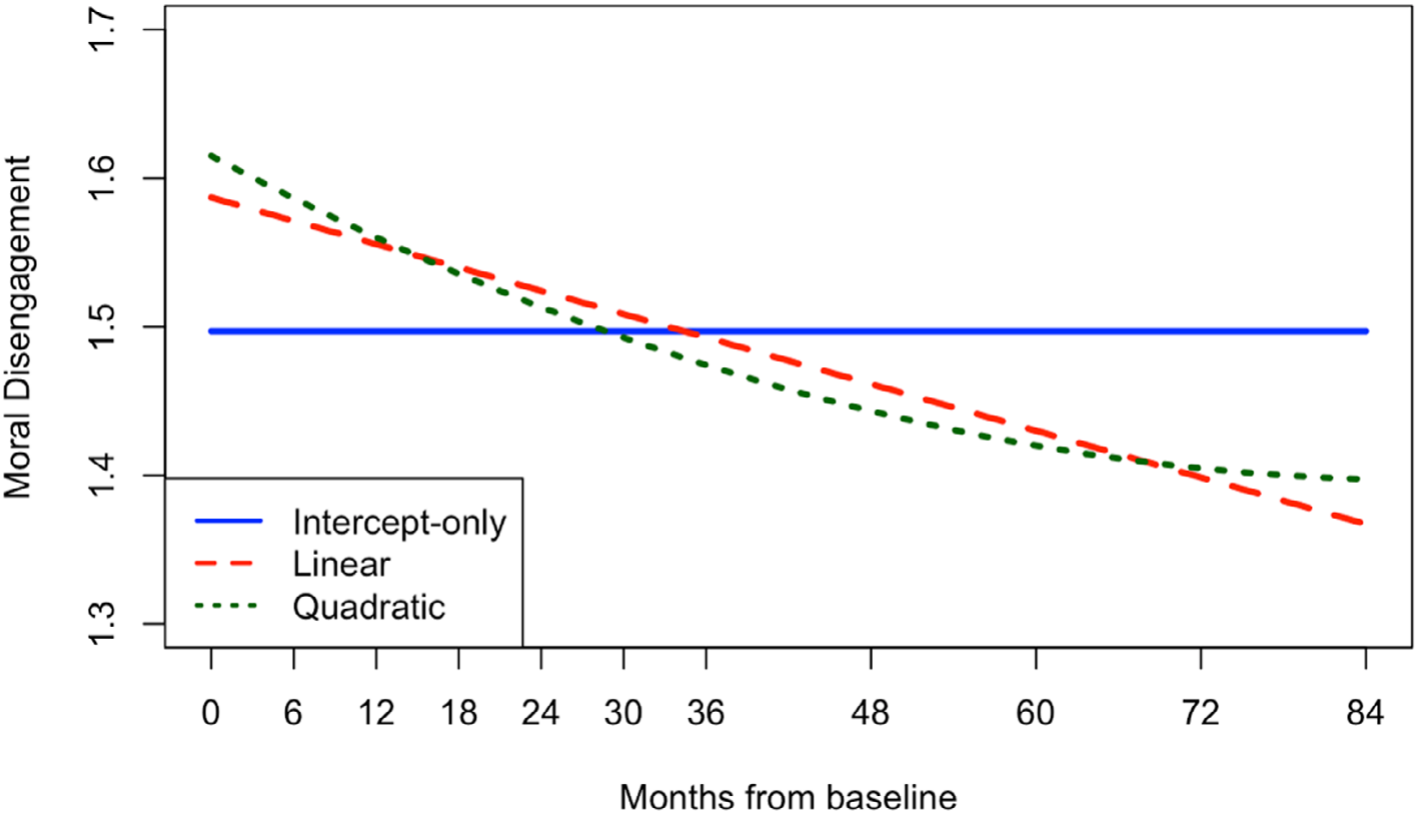
Unconditional latent growth curves of moral disengagement.

The unconditional latent growth curve models revealed that, while linear growth adequately fit the data for PP, PR, and MD, quadratic models demonstrated stronger fit indices and significant curvature (i.e., mean quadratic factors differed from 0; see Tables 2 and 3). On average, MD scores began at 1.62 and declined over time, with the decrease decelerating or flattening across waves (i.e., drop starts to plateau as most youth reach young adulthood; all growth factors *p* < .001). Although consistent with normative development, the timing and magnitude of the deceleration (i.e., steepness of upward bend) suggest a potential premature cessation of growth, occurring before young adulthood (waves 7–8 where mean ages are 19–20) rather than gradually through it (waves 9–11 where mean ages are 21–23; see Figure 4). Similarly, average PR scores started at 3.13 and decreased with a deceleration around the same time (all growth factors *p* < .001). However, in this sample, PR scores surprisingly increased in the final waves (see Figure 3), marking a clear deviation from normative development that would expect reward sensitivity and peer influence to diminish into adulthood. Lastly, youth started with an average PP score of 4.97 that increased into adulthood, though also with a significant premature deceleration around wave 8 (intercept and slope factors *p* < .001, quadratic factor *p* = .013; see Figure 2).

**TABLE 2:** Fit statistics of unconditional growth trajectories across 11 waves (*N* = 1170).

	*X* ^2^	dF	AIC	BIC	SABIC	CFI	TLI	RMSEA	SRMR
**Perception of punishment**									
Intercept only	633.58**	65	43684.80	43745.57	43707.46	0.846	0.870	0.086	0.003
Linear	203.60**	62	43190.60	43266.57	43218.92	0.962	0.966	0.044	0.051
Quadratic	115.76**	58	43095.97	43192.20	43131.85	0.984	0.985	0.029	0.029
**Perception of rewards**									
Intercept only	977.35**	74	39231.10	39246.30	39236.77	0.757	0.819	0.102	0.111
Linear	490.41**	71	38644.78	38675.17	38656.11	0.887	0.913	0.071	0.100
Quadratic	235.55**	67	38341.27	38391.92	38360.15	0.955	0.963	0.046	0.056
**Moral disengagement**									
Intercept only	1097.03**	65	4635.50	4696.28	4658.17	0.723	0.766	0.116	0.158
Linear	317.64**	62	3551.71	3627.68	3580.03	0.931	0.939	0.059	0.106
Quadratic	157.72**	58	3337.64	3433.87	3373.52	0.973	0.975	0.038	0.065

*Note*: **p < .01.

Abbreviations: *X* ^2^, Chi‐square value; df, degrees of freedom.

**TABLE 3:** Estimates of unconditional quadratic growth trajectories across 11 waves (*N* = 1170).

	Growth factor	Estimate	SE	Variance	SE
Perception of punishment	Intercept	4.97**	0.05	1.85**	0.11
	Linear	0.78**	0.13	6.40**	1.00
	Quadratic	−0.21*	0.08	2.20**	0.44
Perception of rewards	Intercept	3.13**	0.04	1.25**	0.08
	Linear	−1.11**	0.11	5.78**	0.70
	Quadratic	0.57**	0.07	2.39**	0.32
Moral disengagement	Intercept	1.62**	0.01	0.08**	0.01
	Linear	−0.29**	0.02	0.28**	0.04
	Quadratic	0.10**	0.02	0.11**	0.02

*Note*: **p < .01, *p < .05.

Abbreviation: SE, standard error.

In terms of covariances, consistent with regression‐to‐the‐mean effects, youth with higher baseline MD and PR scores tended to have steeper decreases in their respective trajectories, and those with higher starting PP values showed less rise over time. Plus, sharper declines in MD and PR and increases in PP were associated with greater deceleration in growth. Still, significant variance existed across participants in their starting points and rates of change (i.e., intercept, linear, quadratic factors all *p* < .001), indicating that not everyone began with the same levels of PP, PR, and MD or had those levels change in the same way (e.g., some experience less growth). These individual differences suggest that some trajectories may confer greater risk for offending than others. However, these latent growth curve models cannot account for concurrent change in the other variables over time (e.g., how change in PP and PR relates to change in MD). Thus, we next estimated an ALT model to examine interrelations among the three variables across time.

#### Autoregressive components

**FIGURE 5:**
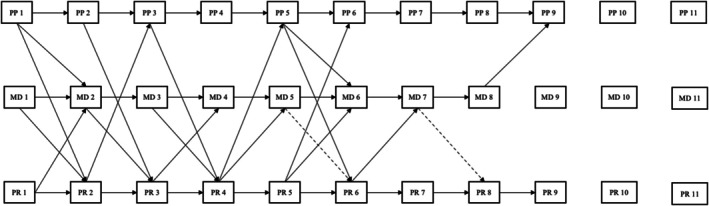
Significant autoregressive and cross‐lagged components in the ALT model. MD, moral disengagement; pp, perception of punishment for crime; PR, perception of rewards for crime; solid lines: *p* < .05; dashed lines: *p* = .05; only significant regressions in the autoregressive and cross‐lagged components of the autoregressive latent trajectory model are shown. Correlations, nonsignificant paths, and the growth components are omitted to improve figure clarity.

In the ALT model, we estimated autoregressive paths to assess the stability of each cognitive process over time after accounting for the latent growth. As depicted in Table 4 and Figure 5, fluctuations in PP, PR, and MD at one wave significantly predicted the same variable at the next wave, but the effect was limited to earlier periods (through wave 9 for PP and PR, and wave 8 for MD). In other words, while there was stability in the processes for earlier waves (i.e., someone's level of PP, PR, and MD at one wave reliably predicted their level at the next), this effect diminished as participants became young adults. Coupled with the observed decelerations in growth around the same time (Figures 2, 3, 4), there appears to be a developmental shift across the three cognitive processes as teens transition into young adults. While some gradual plateauing is normative during this life stage, the abrupt halt of growth across risk‐perception processes may reflect disrupted or premature stabilizations in this justice‐involved sample. Nevertheless, the parallel patterns of growth across these developmental stages may point to a shared neuro‐psychosocial mechanism influencing the three cognitive processes jointly, though further research will need to more thoroughly investigate this possibility.

**TABLE 4:** Autoregressive and cross‐lagged components of the autoregressive latent trajectory model.

Regression	Std. Est. (SE)	Regression	Std. Est. (SE)	Regression	Std. Est. (SE)
**Predicting perceptions of punishment for crime**					
PP1→PP2	0.08* (0.04)	MD1→PP2	0.22 (0.19)	PR1→PP2	−0.04 (0.06)
PP2→PP3	0.14** (0.03)	MD2→PP 3	0.18 (0.15)	PR 2→PP 3	−0.12** (0.04)
PP3→PP4	0.14** (0.03)	MD3→PP 4	0.01 (0.13)	PR 3→PP 4	−0.07 (0.04)
PP4→PP5	0.14** (0.03)	MD4→PP 5	0.01 (0.13)	PR 4→PP 5	−0.08* (0.04)
PP5→PP6	0.17** (0.03)	MD5→PP 6	−0.13 (0.13)	PR 5→PP 6	−0.08* (0.04)
PP6→PP7	0.11** (0.03)	MD6→PP 7	−0.14 (0.14)	PR 6→PP 7	−0.05 (0.04)
PP7→PP8	0.10** (0.03)	MD7→PP 8	−0.17 (0.14)	PR 7→PP 8	−0.05 (0.04)
PP8→PP9	0.07* (0.03)	MD8→PP 9	−0.31* (0.15)	PR 8→PP 9	0.01 (0.04)
PP9→PP10	0.02 (0.04)	MD9→PP 10	−0.32 (0.17)	PR 9→PP 10	0.05 (0.04)
PP10→PP11	−0.11 (0.07)	MD10→PP 11	−0.09 (0.31)	PR 10→PP 11	0.06 (0.07)
**Predicting perceptions of rewards for crime**					
PR1→PR2	0.10* (0.05)	PP1→PR2	−0.08** (0.01)	MD1→PR2	0.38* (0.16)
PR2→PR3	0.10* (0.04)	PP2→PR3	−0.07** (0.02)	MD2→PR3	0.36** (0.14)
PR3→PR4	0.13** (0.04)	PP3→PR4	−0.04* (0.02)	MD3→PR4	0.26* (0.11)
PR4→PR5	0.19** (0.04)	PP4→PR5	−0.02 (0.02)	MD4→PR5	0.14 (0.12)
PR5→PR6	0.18** (0.04)	PP5→PR6	−0.05** (0.02)	MD5→PR6	**0.24^ (0.12)**
PR6→PR7	0.18** (0.04)	PP6→PR7	−0.02 (0.02)	MD6→PR7	0.16 (0.14)
PR7→PR8	0.08* (0.03)	PP7→PR8	−0.01 (0.02)	MD7→PR8	**0.26^ (0.13)**
PR8→PR9	0.08* (0.04)	PP8→PR9	0.01 (0.02)	MD8→PR9	0.14 (0.13)
PR9→PR10	0.09 (0.05)	PP9→PR10	0.00 (0.03)	MD9→PR10	−0.15 (0.16)
PR10→PR11	−0.02 (0.09)	PP10→PR11	0.07 (0.05)	MD10→PR11	−0.34 (0.29)
**Predicting moral disengagement**					
MD1→MD2	0.12** (0.04)	PP1→MD2	0.01* (0.01)	PR1→MD2	0.04** (0.01)
MD2→MD3	0.16** (0.03)	PP2→MD3	0.01 (0.01)	PR2→MD3	0.01 (0.01)
MD3→MD4	0.14** (0.03)	PP3→MD4	0.00 (0.01)	PR3→MD4	0.03** (0.01)
MD4→MD5	0.16** (0.03)	PP4→MD5	−0.00 (0.00)	PR4→MD5	0.02* (0.01)
MD5→MD6	0.17** (0.03)	PP5→MD6	−0.01* (0.00)	PR5→MD6	0.01* (0.01)
MD6→MD7	0.12** (0.03)	PP6→MD7	−0.00 (0.01)	PR6→MD7	0.01* (0.01)
MD7→MD8	0.09** (0.03)	PP7→MD8	0.00 (0.01)	PR7→MD8	0.00 (0.01)
MD8→MD9	0.03 (0.03)	PP8→MD9	0.00 (0.01)	PR8→MD9	0.01 (0.01)
MD9→MD10	0.01 (0.04)	PP9→MD10	−0.00 (0.01)	PR9→MD10	−0.00 (0.01)
MD10→MD11	0.01 (0.08)	PP10→MD11	−0.00 (0.01)	PR10→MD11	−0.02 (0.01)

*Note*: **p < .01, *p < .05, ^*p* = .05; bolded cells are significant.

Abbreviations: MD#, moral disengagement score at wave #; PP#, perceptions of punishment for crime at wave #; PR#, perceptions of rewards for crime at wave #; SE, standard error; Std. Est., standardized estimate.

### Punishment, reward, and moral disengagement: Joint growth

Further evidence of PP, PR, and MD's developmental intertwinement is in Table S1, where all variables are significantly correlated within and across waves (i.e., both contemporaneous and lagged associations). However, the strengths of the relationships vary across time (e.g., less strongly correlated at later waves with longer recall periods).

#### Joint latent growth curves

The MD trajectory was associated with both the PP and PR trajectories, which were unexpectedly unassociated with each other (see Table 5). Adolescents with higher initial MD scores also started with lower PP and higher PR scores. While those youth generally redressed their PP scores with steeper increases in scores and less deceleration across time, this compensatory pattern was not observed for PR. Instead, slower declines and greater deceleration in MD scores were linked to similarly slow and decelerated decreases in PR. Together, these results suggest that MD codevelops differently with PP and PR, such that MD scores in middle adolescence are associated with the subsequent growth of PP, whereas drops and decelerations in MD into adulthood track more closely with PR changes across that time. This divergence may reflect the shared reliance of MD and PR on earlier‐developing limbic reward systems and the impact of MD on the later maturing prefrontal regions associated with long‐term risk evaluation.

**TABLE 5:** Unstandardized covariance of growth factors within the ALT.

	Perception of punishment covariance (SE)			Perception of rewards covariance (SE)			Moral disengagement covariance (SE)		
	Int.	Lin.	Quad.	Int.	Lin.	Quad.	Int.	Lin.	Quad.
**Perception of punishment**									
Intercept	–	−0.78 (0.44)	0.55 (0.32)	−0.07 (0.11)	−0.41 (0.28)	0.14 (0.21)	−0.10** (0.03)	0.13* (0.06)	−0.12** (0.05)
Linear	–	–	−2.07* (0.92)	0.09 (0.27)	−0.35 (0.70)	0.34 (0.49)	0.08 (0.06)	−0.21 (0.15)	0.14 (0.10)
Quadratic	–	–	–	−0.29 (0.19)	0.36 (0.49)	−0.44 (0.37)	−0.05 (0.04)	0.13 (0.10)	−0.10 (0.08)
**Perception of rewards**									
Intercept	–	–	–	–	−0.70* (0.28)	0.31 (0.20)	0.11** (0.02)	−0.10 (0.06)	0.06 (0.04)
Linear	–	–	–	–	–	−1.98** (0.62)	−0.06 (0.05)	0.41** (0.15)	−0.26** (0.11)
Quadratic	–	–	–	–	–	–	0.04 (0.04)	−0.26* (0.11)	0.21** (0.08)
**Moral disengagement**									
Intercept	–	–	–	–	–	–	–	−0.03 (0.02)	0.01 (0.01)
Linear	–	–	–	–	–	–	–	–	−0.09** (0.03)
Quadratic	–	–	–	–	–	–	–	–	–

*Note*: **p < .01, *p < .05.

Abbreviations: Int, intercept factor; Lin, linear factor; Quad, quadratic factor; SE, standard error.

#### Cross‐lag components

Finally, the cross‐lag components revealed bidirectional relationships between MD and PR, as well as PP and PR, such that fluctuations in one variable predicted corresponding (MD and PR) or opposing (PP and PR) changes in the other at the next wave (see Table 4 and Figure 5). In other words, shifts in how individuals viewed MD and PP impacted their perceived rewards at the next time point, which then influenced their future MD and PP. These dynamic and reciprocal within‐person effects emerged on top of stable between‐person relationships, highlighting the evolving interplay that shapes individual trajectories over time. However, these effects also diminished in magnitude and lost significance by the seventh and sixth waves, respectively. In contrast, the relationship between MD and PP was less clear across development, with weak and inconsistent predictive findings (e.g., positive effect of PP on MD from wave 1 to 2 but negative effect from waves 5 to 6; MD only negatively predicting PP from wave 8 to 9).

Post hoc analyses of individual parameters (e.g., just wave 8 MD predicting wave 9 PP) supported most ALT findings but revealed a negative, not positive, association between PP and MD from wave 1 to 2. This discrepancy may reflect a modeling artifact (e.g., Heywood case) or the influence of unmeasured confounding factors (e.g., arrests). Full results are available upon request. Nevertheless, although the MD‐PP link warrants further investigation, the totality of these results corroborates the hypothesis that cyclical patterns between these three variables may distort healthy risk perception development and consequently increase offending vulnerability.

## DISCUSSION

Guided by rational, moral, and neurological theories of crime, we used a novel three‐variable ALT model to illustrate MD's role in risk perception. Based on the tripartite framework, we investigated individuals' expected utility (PR), external disutility (PP), and internal disutility (guilt controlled by MD) across peak crime ages. By separating within‐ and between‐individual differences, we identified stable traits and time‐sensitive shifts that increase the risk for life‐course offending. This distinction revealed that PP, PR, and MD bidirectionally influence one another in ways that can amplify risks, and that framing them as an integrated mechanism offers a more nuanced, real‐world account as to why youth and young adults choose to offend. These insights inform the timing and targets of developmentally tailored programs to more effectively disrupt maladaptive cognitive patterns among youth already engaging in risky behaviors.

### Punishment, reward, and moral disengagement: Individual growth

Broadly, justice‐involved individuals may be at increased risk for engaging in crime due to premature decelerations in their PP, PR, and MD development during adolescence that result in underdeveloped risk perception mechanisms. Rather than exhibiting gradual, stable growth through young adulthood, we observed early and steep developmental plateaus in these processes alongside a loss of stability within the variables (i.e., variables stopped predicting themselves). This temporal pattern suggests that developmental delays in risk‐related cognition may lead to the inconsistent use of the tripartite decision‐making process across opportunities in adulthood. As such, interventions should teach and practice moral education, problem‐solving skills to replace MD, and realistic portrayals of the consequences and social appeal of crime. While such efforts may accelerate positive change in early adolescence, young adults may also require more advanced booster sessions to maintain progress and prevent cognitive and moral stagnation.

### Punishment, reward, and moral disengagement: Joint growth

Beyond parallel growth similarities, the three processes influenced each other across development. Consistent with Neo‐Kohlbergian theories (Rest et al., 1999), reductions in MD and PR coincided with increases in PP, suggesting a normative shift toward internalized morality and balanced risk perception. However, persistently elevated MD and PR seemed to inhibit PP growth, potentially disrupting healthy cognitive and moral maturation. This aligns with the dual systems theory, where disrupted neural integration between reward‐driven limbic regions and regulatory prefrontal cortex may sustain these problematic patterns (Galvan et al., 2007). Indeed, the weakening relationships in later waves suggest that disrupted neural connectivity during adolescence may not only reinforce harmful cycles but also prematurely halt growth, leaving youth cognitively vulnerable just as adult responsibilities mount. Importantly, interventions will need to recognize and respond to the distinct, evolving relationships of PP, PR, and MD.

Specifically, MD and PR growth cooccurs across waves, such that both the starting levels and rates of change in one process are linked to those of the other. Coupled with their bidirectional predictive relationship, this suggests that youth who morally disengage to avoid guilt may view crime as more rewarding, which simultaneously reinforces MD as a coping tool to justify unethical behaviors. Over time, this may form a self‐reinforcing cycle that disrupts neurological growth and the normative decline of both processes. Indeed, the strength of this relationship fades in late adolescence, when the decelerations begin, raising the possibility that these mutually reinforcing patterns may contribute to the premature plateaus in risk perception. However, future research will need to further explore the temporal sequencing of these effects.

On the other hand, although higher adolescent MD scores were associated with lower PP across waves, changes in MD were not consistently linked to changes in PP. This reflects Neo‐Kohlbergian theories, such that moral development shifts from an external focus on avoiding punishment to more complex moral reasoning based on social norms and principles. However, we found that higher MD in later waves may dampen the expected increase in PP from growing sensitivity to external consequences and the move toward internalized moral standards. Notably, the timing of this stagnation also aligned with the decelerations in growth for both MD and PP and thus may be another contributing factor to the overall curtailed maturation of risk perception. Alternatively, results may be confounded by heterogeneous growth that obscures clearer group patterns (i.e., subgroups maturing in different ways). Regardless, the stronger MD‐PR relationship in earlier waves, and MD‐PP in later ones, illustrate Neo‐Kohlbergian and neurological shifts in cognitive processes, such that adolescents appear to justify behaviors to seek reward, while young adults rationalize their actions to minimize perceived consequences.

Lastly, although the PP and PR growth trajectories were not significantly associated, adolescents who expected less punishment were more inclined to perceive benefits for crimes at the next wave, and those who did were then less likely to expect consequences. In this sense, lower perceived risks may inflate the perceived rewards of a crime, fostering overconfidence and a feeling of invincibility regarding the likelihood and costs of consequences. Since these findings were not reflected in the broader growth trajectories, they may be especially salient during early adolescence. However, as cognitive capacities mature with brain development (e.g., impulse control, planning), PP tends to increase and PR decrease, allowing young adults to better weigh the outcomes of crime. Importantly, understanding how these early reciprocal distortions contribute to long‐term risk perception highlights key windows and targets for intervention.

### Implications

While PP, PR, and MD function as independent processes within a shared risk perception framework, the developmental networks between MD to PP, MD to PR, and PP to PR differ. This means that interventions should not only target each process individually, but also address the dyadic relationships (e.g., MD–PR) and their combined influence. Effective programs will need to recalibrate distorted views of crime's consequences and rewards while fostering stable moral reasoning and problem‐solving skills that are less influenced by the immediate rewards and more by the potential punishments. For instance, beyond discouraging MD and reinforcing moral choices, interventions should help adolescents reduce exaggerated reward perceptions and encourage young adults to recognize the potential harm of their actions. Indeed, it may be especially useful to teach impulse control, emotional regulation, and cognitive strategies like self‐talk to ensure that at‐risk youth pause to evaluate all aspects of a decision before offending.

Notably, treatment should prioritize PR, as scores increased at the final waves and showed strong bidirectional relationships with both MD and PP. Since our measure emphasizes the social and personal rewards of crime, this rise may reflect a failure to undergo normative decline in peer influence or an underdeveloped reward processing sensitivity (Steinberg, 2008). For example, justice‐involved youth who remain embedded in peer groups that normalize or glamorize offending may reinforce their distorted perceptions of crime's social benefits. This stresses the importance of reentry programs that connect youth with prosocial community influences that support cognitive reframing strategies to challenge faulty social perceptions, reduce the appeal of crime‐related rewards, and naturally recalibrate broader risk perception.

Fortunately, several evidence‐based programs emphasize our recommendations. For instance, moral reconation therapy has been shown to enhance moral reasoning and reduce recidivism by addressing cognitive distortions and fostering ethical decision‐making (Ferguson & Wormith, 2013). Alternatively, Thinking for a Change integrates cognitive restructuring, social skills, and problem‐solving training to target criminogenic thinking and the assessment of punishments and rewards (Lowenkamp et al., 2009). Further, Aggression Replacement Training focuses on social skills, anger control, and moral reasoning to reduce antisocial behaviors and MD by promoting prosocial behavior and moral judgments (Goldstein et al., 1998). Although these do not independently cover all of our suggestions, adapting them to simultaneously address the integrated developmental trajectories of PP, PR, and MD identified in this study may better disrupt maladaptive risk‐taking patterns and support healthier adolescent development.

Regardless, interventions designed to disrupt these harmful cycles will be most effective if implemented earlier in adolescence, ideally before the transition to young adulthood, as it may be a critical period when developmental decelerations become difficult to reverse. Notably, our recommendation for booster sessions to sustain growth during that time challenges traditional approaches that favor short‐term, front‐loaded interventions based on the assumption that early correction is sufficient to alter long‐term change (Greenwood et al., 1998). Nevertheless, while our overall interpretations emphasize risk due to the nature of our sample, the bidirectional relationships could also be a protective dynamic that promotes healthy development. That is, although higher MD scores are associated with higher PR and lower PP, the reverse is also true: lower MD is linked to lower PR and higher PP. This suggests that early intervention could convert harmful feedback loops into reinforcing protective cycles that accelerate normative growth in risk perception and moral reasoning while reducing long‐term offending.

### Strengths, limitations, and future studies

This study employed a novel longitudinal approach (i.e., three‐variable ALT model) to examine how PP, PR, and MD processes interrelate across the transition to adulthood. Modeling all three cognitions together allowed us to glean rich, developmentally nuanced insights that advance knowledge on how each process independently evolves and how their interplay across development relates to peak periods of offending. This more holistic and ecological perspective highlights how thoughts surrounding risky decision making can go awry and increase criminal behaviors. Notably, we identified specific nonnormative developmental trends and proposed targeted, actionable strategies for improving crime prevention and intervention by focusing on the cognitive roots of offending. However, our study has limitations that warrant addressing.

First, due to the scarcity of longitudinal studies on these processes, our assumption that development goes awry in justice‐involved youth is primarily grounded in theories of normative development rather than direct comparisons to community samples. Additionally, we could not converge ALT models with data aligned by age or 12‐month intervals, limiting interpretability and accountability for the study's procedural effects (i.e., 6‐month vs. 12‐month intervals). As a result, our interpretations reflect broader age groups rather than precise ages and are limited to the average age at each wave (16 to 23). Therefore, the observed premature decelerations could represent delayed growth that resumes later in adulthood rather than terminating early. Similarly, we could not distinguish whether the developmental plateaus represent capacity limitations or the attainment of adequate growth, and thus future studies will need to identify benchmarks or cut‐off scores for these processes to better determine who may be at heightened risk.

Furthermore, our results are limited to justice‐involved boys from the U.S.A., so other groups (e.g., girls, youth from the community or other countries) may value the processes differently based on cultural norms (e.g., punitive nations emphasize consequences). It also remains unclear how PP, PR, and MD moderate or mediate one another in leading to varied outcomes (e.g., substance use, empathy), or how environmental and cognitive influences (e.g., peer delinquency, executive functioning) impact the individual or joint processes. Notably, youths' reactions to confounding experiences like arrests or offending may account for some of our findings. For instance, getting arrested may increase PP and reduce MD and PR, while repeated offending could reinforce PR and MD when successful or decrease PP if consequences are minimal. Likewise, impulsivity may override decision‐making altogether (Pogarsky, 2002). Future work should examine these processes alongside justice involvement, offending, and self‐regulatory capacities to better isolate developmental trajectories from reactive adaptations.

Moreover, because we did not track how specific MD strategies (e.g., moral justification) temporally evolve, it remains unclear whether overall decreases in MD are due to broad declines across strategies or the fading of specific ones (e.g., dehumanization). Consequently, it is unclear how MD's eight multifaceted mechanisms are intertwined with each other and broader risk perception mechanisms. Thus, future work should disentangle the individual components to identify which strategies are most developmentally persistent and strongly linked to offending and disengagement. Finally, our study would benefit from complementary longitudinal research examining the growth of limbic and prefrontal regions and the connectivity between them. This would help determine whether incomplete integration of these systems underlies the developmental patterns we observed in risk evaluation, reward sensitivity, and moral reasoning.

## CONCLUSION

By examining the joint growth of PP, PR, and MD in a sample of justice‐involved youth, we identified potential abnormalities in risk perception development that may be leading to increased risk for committing crimes. Notably, we found evidence that the three independent processes share a broader underlying mechanism, with unique networks that connect MD to PP, MD to PR, and PP to PR across development. However, these relationships can be harmful and can negatively impact the normative growth of risk perception, and thus intervention efforts will need to focus on the three processes independently, as duads, and as a broader group to be most effective. Specifically, interventions should target MD early in adolescence to recalibrate perceptions of both risk and reward and prevent the reinforcement of maladaptive cognitive patterns. Importantly, this work will need to occur early, as the transition from late adolescence to young adulthood appears to be a critical developmental period for risk perception.

## FUNDING INFORMATION

No funding was received for this secondary data analysis.

## CONFLICT OF INTEREST STATEMENT

The authors have no conflict of interest to disclose.

## PATIENT CONSENT STATEMENT

Parental or participant consent was obtained when data was originally collected. This consent included permission for researchers to later conduct secondary data analyses like the current study. Information on the study procedures and measures can be found in Schubert et al., 2004 or at pathwaysstudy.pitt.edu.

## Supporting information


Data S1.

## Data Availability

The data that support the findings of this study are openly available in ICPSR at https://icpsr.umich.edu/web/NAHDAP/studies/29961.
