# Supplementary material for: Cognitive loopholes of crime: Mapping the Codevelopment of moral disengagement within perceptions of risks and rewards
Source: J Res Adolesc. 2025 Jul 29;35(3):e70056. doi: 10.1111/jora.70056 (PMC12305401; doi:10.1111/jora.70056)
Supplement: Supplementary file 1 — Data S1. [file JORA-35-0-s001.docx]

**APPENDIX B: SUPPLEMENTAL MATERIAL**

| Supplemental Table 1 | | | | | | | | | | | | | | | | | | | | | | | | | | | | | | | | | |
| --- | --- | --- | --- | --- | --- | --- | --- | --- | --- | --- | --- | --- | --- | --- | --- | --- | --- | --- | --- | --- | --- | --- | --- | --- | --- | --- | --- | --- | --- | --- | --- | --- | --- |
| *Pairwise Correlations Across Waves* | | | | | | | | | | | | | | | | | | | | | | | | | | | | | | | | | |
|  | MD 1 | PP 1 | PR 1 | MD 2 | PP 2 | PR 2 | MD 3 | PP 3 | PR 3 | MD 4 | PP 4 | PR 4 | MD 5 | PP 5 | PR 5 | MD 6 | PP 6 | PR 6 | MD 7 | PP 7 | PR 7 | MD 8 | PP 8 | PR 8 | MD 9 | PP 9 | PR 9 | MD 10 | PP 10 | PR 10 | MD 11 | PP 11 | PR 11 |
| MD 1 | 1.00 |  |  |  |  |  |  |  |  |  |  |  |  |  |  |  |  |  |  |  |  |  |  |  |  |  |  |  |  |  |  |  |  |
| PP 1 | -0.17** | 1.00 |  |  |  |  |  |  |  |  |  |  |  |  |  |  |  |  |  |  |  |  |  |  |  |  |  |  |  |  |  |  |  |
| PR 1 | 0.47** | -0.18** | 1.00 |  |  |  |  |  |  |  |  |  |  |  |  |  |  |  |  |  |  |  |  |  |  |  |  |  |  |  |  |  |  |
| MD 2 | 0.59** | -0.11** | 0.41** | 1.00 |  |  |  |  |  |  |  |  |  |  |  |  |  |  |  |  |  |  |  |  |  |  |  |  |  |  |  |  |  |
| PP 2 | -0.16** | 0.50** | -0.17** | -0.21** | 1.00 |  |  |  |  |  |  |  |  |  |  |  |  |  |  |  |  |  |  |  |  |  |  |  |  |  |  |  |  |
| PR 2 | 0.36** | -0.14** | 0.56** | 0.47** | -0.15** | 1.00 |  |  |  |  |  |  |  |  |  |  |  |  |  |  |  |  |  |  |  |  |  |  |  |  |  |  |  |
| MD 3 | 0.51** | -0.15** | 0.32** | 0.62** | -0.15** | 0.39** | 1.00 |  |  |  |  |  |  |  |  |  |  |  |  |  |  |  |  |  |  |  |  |  |  |  |  |  |  |
| PP 3 | -0.19** | 0.48** | -0.20** | -0.19** | 0.54** | -0.19** | -0.23** | 1.00 |  |  |  |  |  |  |  |  |  |  |  |  |  |  |  |  |  |  |  |  |  |  |  |  |  |
| PR 3 | 0.28** | -0.10** | 0.49** | 0.40** | -0.15** | 0.57** | 0.42** | -0.17** | 1.00 |  |  |  |  |  |  |  |  |  |  |  |  |  |  |  |  |  |  |  |  |  |  |  |  |
| MD 4 | 0.48** | -0.10** | 0.32** | 0.55** | -0.16** | 0.38** | 0.63** | -0.19** | 0.40** | 1.00 |  |  |  |  |  |  |  |  |  |  |  |  |  |  |  |  |  |  |  |  |  |  |  |
| PP 4 | -0.10** | 0.41** | -0.13** | -0.14** | 0.48** | -0.12** | -0.14** | 0.58** | -0.15** | -0.20** | 1.00 |  |  |  |  |  |  |  |  |  |  |  |  |  |  |  |  |  |  |  |  |  |  |
| PR 4 | 0.27** | -0.06* | 0.43** | 0.34** | -0.08* | 0.52** | 0.38** | -0.14** | 0.59** | 0.49** | -0.16** | 1.00 |  |  |  |  |  |  |  |  |  |  |  |  |  |  |  |  |  |  |  |  |  |
| MD 5 | 0.47** | -0.12** | 0.36** | 0.53** | -0.14** | 0.35** | 0.58** | -0.20** | 0.34** | 0.63** | -0.18** | 0.40** | 1.00 |  |  |  |  |  |  |  |  |  |  |  |  |  |  |  |  |  |  |  |  |
| PP 5 | -0.16** | 0.41** | -0.19** | -0.19** | 0.42** | -0.15** | -0.16** | 0.50** | -0.11** | -0.19** | 0.53** | -0.17** | -0.23** | 1.00 |  |  |  |  |  |  |  |  |  |  |  |  |  |  |  |  |  |  |  |
| PR 5 | 0.28** | -0.11** | 0.42** | 0.32** | -0.10** | 0.47** | 0.32** | -0.14** | 0.56** | 0.39** | -0.15** | 0.61** | 0.45** | -0.17** | 1.00 |  |  |  |  |  |  |  |  |  |  |  |  |  |  |  |  |  |  |
| MD 6 | 0.46** | -0.15** | 0.33** | 0.50** | -0.17** | 0.33** | 0.56** | -0.18** | 0.33** | 0.60** | -0.18** | 0.38** | 0.67** | -0.22** | 0.41** | 1.00 |  |  |  |  |  |  |  |  |  |  |  |  |  |  |  |  |  |
| PP 6 | -0.15** | 0.40** | -0.16** | -0.18** | 0.40** | -0.18** | -0.19** | 0.46** | -0.17** | -0.21** | 0.49** | -0.18** | -0.23** | 0.56** | -0.21** | -0.29** | 1.00 |  |  |  |  |  |  |  |  |  |  |  |  |  |  |  |  |
| PR 6 | 0.25** | -0.08* | 0.36** | 0.29** | -0.11** | 0.43** | 0.31** | -0.15** | 0.48** | 0.37** | -0.16** | 0.58** | 0.39** | -0.23** | 0.62** | 0.47** | -0.19** | 1.00 |  |  |  |  |  |  |  |  |  |  |  |  |  |  |  |
| MD 7 | 0.42** | -0.10** | 0.29** | 0.45** | -0.12** | 0.30** | 0.49** | -0.19** | 0.30** | 0.52** | -0.16** | 0.34** | 0.58** | -0.17** | 0.38** | 0.65** | -0.19** | 0.39** | 1.00 |  |  |  |  |  |  |  |  |  |  |  |  |  |  |
| PP 7 | -0.10** | 0.39** | -0.14** | -0.14** | 0.42** | -0.19** | -0.14** | 0.48** | -0.16** | -0.15** | 0.50** | -0.14** | -0.17** | 0.53** | -0.17** | -0.21** | 0.55** | -0.19** | -0.22** | 1.00 |  |  |  |  |  |  |  |  |  |  |  |  |  |
| PR 7 | 0.19** | -0.13** | 0.35** | 0.27** | -0.12** | 0.41** | 0.25** | -0.18** | 0.45** | 0.32** | -0.21** | 0.51** | 0.36** | -0.23** | 0.56** | 0.36** | -0.24** | 0.60** | 0.43** | -0.24** | 1.00 |  |  |  |  |  |  |  |  |  |  |  |  |
| MD 8 | 0.40** | -0.13** | 0.30** | 0.41** | -0.14** | 0.30** | 0.49** | -0.20** | 0.29** | 0.51** | -0.17** | 0.28** | 0.53** | -0.18** | 0.34** | 0.58** | -0.21** | 0.35** | 0.59** | -0.18** | 0.33** | 1.00 |  |  |  |  |  |  |  |  |  |  |  |
| PP 8 | -0.11** | 0.31** | -0.13** | -0.16** | 0.37** | -0.13** | -0.14** | 0.41** | -0.15** | -0.14** | 0.44** | -0.15** | -0.15** | 0.48** | -0.16** | -0.15** | 0.48** | -0.14** | -0.20** | 0.56** | -0.26** | -0.20** | 1.00 |  |  |  |  |  |  |  |  |  |  |
| PR 8 | 0.19** | -0.20** | 0.33** | 0.20** | -0.14** | 0.33** | 0.25** | -0.15** | 0.41** | 0.31** | -0.19** | 0.40** | 0.34** | -0.22** | 0.46** | 0.34** | -0.24** | 0.49** | 0.36** | -0.22** | 0.54** | 0.46** | -0.21** | 1.00 |  |  |  |  |  |  |  |  |  |
| MD 9 | 0.33** | -0.07* | 0.25** | 0.35** | -0.08* | 0.27** | 0.40** | -0.13** | 0.25** | 0.43** | -0.08* | 0.29** | 0.45** | -0.12** | 0.26** | 0.49** | -0.17** | 0.28** | 0.53** | -0.17** | 0.34** | 0.57** | -0.18** | 0.38** | 1.00 |  |  |  |  |  |  |  |  |
| PP 9 | -0.11** | 0.32** | -0.17** | -0.20** | 0.39** | -0.15** | -0.17** | 0.44** | -0.18** | -0.22** | 0.43** | -0.17** | -0.21** | 0.44** | -0.15** | -0.20** | 0.46** | -0.16** | -0.22** | 0.49** | -0.24** | -0.25** | 0.54** | -0.23** | -0.23** | 1.00 |  |  |  |  |  |  |  |
| PR 9 | 0.21** | -0.14** | 0.29** | 0.24** | -0.12** | 0.31** | 0.20** | -0.16** | 0.36** | 0.29** | -0.17** | 0.39** | 0.30** | -0.17** | 0.39** | 0.31** | -0.23** | 0.44** | 0.32** | -0.20** | 0.46** | 0.39** | -0.19** | 0.56** | 0.47** | -0.27** | 1.00 |  |  |  |  |  |  |
| MD 10 | 0.33** | -0.10** | 0.24** | 0.36** | -0.12** | 0.26** | 0.41** | -0.15** | 0.29** | 0.45** | -0.18** | 0.30** | 0.49** | -0.12** | 0.32** | 0.51** | -0.17** | 0.30** | 0.54** | -0.20** | 0.34** | 0.60** | -0.19** | 0.37** | 0.56** | -0.19** | 0.37** | 1.00 |  |  |  |  |  |
| PP 10 | -0.13** | 0.29** | -0.14** | -0.21** | 0.37** | -0.18** | -0.21** | 0.43** | -0.21** | -0.20** | 0.46** | -0.20** | -0.23** | 0.44** | -0.18** | -0.23** | 0.49** | -0.19** | -0.22** | 0.48** | -0.23** | -0.25** | 0.51** | -0.25** | -0.24** | 0.56** | -0.24** | -0.27** | 1.00 |  |  |  |  |
| PR 10 | 0.15** | -0.11** | 0.25** | 0.19** | -0.10** | 0.29** | 0.20** | -0.13** | 0.36** | 0.26** | -0.15** | 0.2** | 0.26** | -0.11** | 0.39** | 0.25** | -0.20** | 0.42** | 0.30** | -0.19** | 0.45** | 0.36** | -0.22** | 0.52** | 0.35** | -0.22** | 0.55** | 0.41** | -0.26** | 1.00 |  |  |  |
| MD 11 | 0.33** | -0.08* | 0.23** | 0.36** | -0.12** | 0.26** | 0.34** | -0.17** | 0.24** | 0.41** | -0.13** | 0.24** | 0.44** | -0.12** | 0.24** | 0.43** | -0.17** | 0.22** | 0.47** | -0.16** | 0.26** | 0.51** | -0.18** | 0.33** | 0.50** | -0.2-** | 0.31** | 0.59** | -0.27** | 0.32** | 1.00 |  |  |
| PP 11 | -0.17** | 0.27** | -0.20** | -0.20** | 0.35** | -0.18** | -0.19** | 0.41** | -0.23** | -0.23** | 0.42** | -0.23** | -0.21** | 0.39** | -0.22** | -0.21** | 0.45** | -0.22** | -0.20** | 0.44** | -0.25** | -0.24** | 0.48** | -0.26** | -0.22** | 0.54** | -0.26** | -0.25** | 0.56** | -0.27** | -0.26** | 1.00 |  |
| PR 11 | 0.19** | -0.11** | 0.25** | 0.21** | -0.09** | 0.27** | 0.15** | -0.13** | 0.31** | 0.22** | -0.09** | 0.33** | 0.21** | -0.11** | 0.29** | 0.22** | -0.15** | 0.31** | 0.23** | -0.12** | 0.40** | 0.28** | -0.14** | 0.40** | 0.27** | -0.19** | 0.46** | 0.33** | -0.21** | 0.52** | 0.41** | -0.23** | 1.00 |
| Note: ** p < .01, * p < .05; MD # = Moral Disengagement score at wave #, PP # = Perceived Punishment for Crime score at wave #, PR = Perceived Rewards for Crime score at wave #. | | | | | | | | | | | | | | | | | | | | | | | | | | | | | | | | | |
